# Case Report: Recurrent hypoglycemia due to graft-derived insulinoma in a patient with type 1 diabetes following pancreas-kidney transplantation

**DOI:** 10.3389/frtra.2026.1889705

**Published:** 2026-08-03

**Authors:** Carme Figueredo, Maria Iraola, Marta Aymerich, Neus Villamor, Ivan Archilla, Joana Ferrer-Fàbrega, Antonio J. Amor, Pedro Ventura-Aguiar

**Affiliations:** 1 Endocrinology and Nutrition Department, Hospital Clínic, Barcelona, Spain; 2 Nefrology and Kidney Transplantation Department, Hospital Clínic, Barcelona, Spain; 3 Pathology Department, Hospital Clínic, Barcelona, Spain; 4 Centro de Investigación Biomédica en Red de Diabetes y Enfermedades Metabólicas Asociadas (CIBERDEM), Instituto de Salud Carlos III (ISCIII), Hospital Clínic Barcelona, Madrid, Spain; 5 Institut d'investigacions Biomèdiques August Pi I Sunyer (IDIBAPS), Hospital Clínic Barcelona, Barcelona, Spain; 6 Hepatobiliopancreatic Surgery and Liver and Pancreatic Transplantation Unit, Department of Surgery, Institute Clínic of Digestive and Metabolic Diseases (ICMDiM), Hospital Clínic, Barcelona, Spain; 7 Network for Biomedical Research in Hepatic and Digestive Diseases (CIBEREHD), Barcelona, Spain; 8 Universitat de Barcelona, Barcelona, Spain

**Keywords:** endogenous hyperinsulinism, hypoglycemia, insulinoma, neuroendocrine tumor, simultaneous pancreas-kidney (SPK) transplantation

## Abstract

Hypoglycemia is a frequent finding after simultaneous pancreas–kidney (SPK) transplantation, but its etiology is often multifactorial and challenging to interpret. We report a rare case of late-onset severe hypoglycemia secondary to a neuroendocrine tumor arising in a pancreatic allograft. A 60-year-old man with type 1 diabetes mellitus underwent SPK transplantation in 2007 with immediate graft function and long-term metabolic stability. Ten years post-transplant, partial pancreatic graft dysfunction and progressive vascular complications were noted. In 2024, 17 years after transplantation, the patient developed recurrent, documented hypoglycemic episodes despite withdrawal of glucose-lowering therapy, with fasting plasma glucose values as low as 40 mg/dL and a marked reduction in HbA1c. Biochemical evaluation suggested endogenous hyperinsulinism, with elevated chromogranin A levels. Imaging studies revealed a large hypervascular mass within the pancreatic graft, with high somatostatin receptor expression on 68Ga-DOTA-TOC PET-CT. Histology from biopsy and subsequent total graft pancreatectomy confirmed a well-differentiated grade 2 neuroendocrine tumor with focal insulin expression, consistent with insulinoma. Surgical resection resulted in complete resolution of hypoglycemia. This case highlights the need to consider rare but clinically significant causes of hypoglycemia in SPK recipients and underscores the importance of a comprehensive, multidisciplinary diagnostic approach integrating continuous glucose monitoring, biochemical markers, and advanced imaging.

## 1 Introduction

Simultaneous pancreas–kidney (SPK) transplantation is an established therapeutic option for selected patients with type 1 diabetes mellitus and end-stage kidney disease, providing sustained glycemic control, insulin independence, and improved quality of life (1). Despite favorable long-term metabolic outcomes, disturbances in glucose homeostasis after pancreas transplantation are common, with hypoglycemia reported in up to 50% of recipients. Most episodes are mild, asymptomatic, and attributed to graft-related hyperinsulinemia, impaired counterregulatory responses, or systemic venous drainage of the pancreatic allograft (2).

The diagnostic evaluation of hypoglycemia in SPK recipients is challenging, as interpretation of conventional biochemical tests is limited by the absence of validated reference ranges and by the intrinsic hyperinsulinemic milieu. As a result, hypoglycemia is frequently considered an expected post-transplant phenomenon, potentially delaying recognition of pathological causes (3).

Insulinoma and other functioning pancreatic neuroendocrine tumors have not been previously reported after pancreas transplantation. Herein, we describe the first known case of a functioning insulin-secreting pancreatic neuroendocrine tumor arising in a pancreatic allograft 17 years after SPK transplantation, presenting as severe recurrent hypoglycemia.

## 2 Case description and diagnostic assessment

A 60-year-old white man with a history of T1DM since the age of 12, presenting microvascular complications including proliferative diabetic retinopathy, nephropathy, and peripheral artery disease, underwent simultaneous pancreas–kidney transplantation in 2007. Induction immunosuppression consisted of basiliximab, tacrolimus, mycophenolate, and prednisone. Surgery was uneventful, with immediate graft function of both organs. Maintenance immunosuppression included tacrolimus, mycophenolate, and prednisone according to center standard of care (tacrolimus trough levels 5–7 ng/mL). In 2017, partial pancreatic graft dysfunction was identified during routine follow-up, leading to initiation of DPP-4 inhibitor therapy. Progression of peripheral artery disease required infrapatellar amputation of the right lower limb in 2022.

Two years later, the patient experienced recurrent hypoglycemic episodes despite discontinuation of hypoglycemic agents, as confirmed by continuous glucose monitoring. Hospital admission revealed asymptomatic fasting hypoglycemia, with plasma glucose levels as low as 40 mg/dL. In parallel, HbA1c decreased from 6.5% to 5.0% over the preceding three months.

Laboratory analysis showed elevated chromogranin A levels (622 ng/mL; reference <450 ng/mL), with pharmacological causes of false-positive results excluded. Fasting test results were consistent with endogenous hyperinsulinism, possibly due to insulinoma or non–insulinoma pancreatogenous hypoglycemia (Table 1). Contrast-enhanced CT angiography revealed a hyperenhancing mass within the pancreatic graft measuring 6.9 × 6.5 cm (Figure 1A). 68Ga-DOTA-TOC PET-CT demonstrated high expression of somatostatin receptor subtypes 2 and 5 without evidence of metastatic disease (Figure 1B). Ultrasound-guided biopsy confirmed a well-differentiated grade 2 neuroendocrine tumor with a Ki-67 index of 6%. Immunohistochemistry was positive for chromogranin A and synaptophysin, with focal insulin and glucagon expression, and negative for gastrin and somatostatin.

**Table 1:** Fasting test: results consistent with endogenous hyperinsulinism.

	Glucose (mg/dL)	Insulin (uU/mL)	C-peptide	Proinsulin (pmol/L)	Circulation oral hypoglycemic agent (OHA)	Antibody to insulin (AI) (U/mL)
Reference values	>55	2.21–14	0.4–2.6	<1.6	–	<0.4
Results	42	75	3.4	141.7	No	<0.1

**Figure 1:**
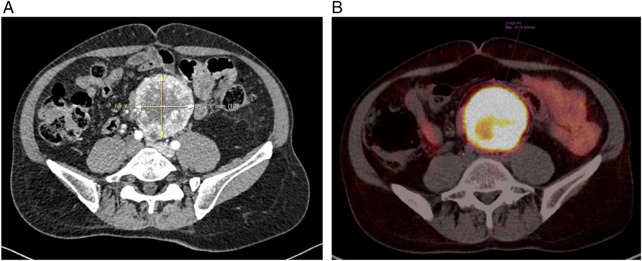
**(A)** CT angiography: it shows a hyperenhancing mass approximately 6.9 × 6.5 cm, located in the head region of the pancreatic graft. It exhibits areas of necrosis and intralesional arterial pseudoaneurysms, suggestive of a neoplastic process. **(B)** PET CT 68Ga DOTA TOC: previously described lesion is observed, consistent with a tumor dependent on the pancreatic graft, showing high expression of somatostatin receptors (types 2 and 5). No findings suggestive of thoracoabdominal extension are noted.

Given suspicion of an insulinoma arising from the pancreatic allograft, total graft pancreatectomy was performed. Pathological examination revealed an 8.5 × 7.6 × 6.5 cm grade 2 neuroendocrine tumor confined to the pancreatic parenchyma, with negative margins, no vascular or perineural invasion, and no lymph node involvement (pT3). Adjacent pancreatic tissue showed gastric-type PanIN with low-grade dysplasia and mild septal fibrosis (<30%).

Postoperatively, hypoglycemic symptoms resolved. Upon discharge, a multiple daily injection insulin regimen with continuous glucose monitoring was reinstated. At 6-month follow-up, imaging showed no recurrence. Microsatellite analysis revealed mixed chimerism, limiting definitive determination of tumor origin.

## 3 Discussion and conclusions

Hypoglycemia in individuals without a diagnosis of diabetes is defined as plasma glucose <55 mg/dL and is considered a relatively rare condition. The presence of Whipple's triad is usually required: (i) symptoms associated with hypoglycemia, (ii) a low plasma glucose concentration (<55 mg/dL in subjects without diabetes), and (iii) symptom relief after normalization of blood glucose levels. Symptoms are classified as adrenergic or neuroglycopenic. If blood glucose falls below 30 mg/dL and no treatment is administered, irreversible neurological damage may occur, and in the worst cases, death.

The diagnosis can sometimes be complex, and several classifications have been proposed to facilitate it. The most widely accepted approach is based on the clinical context (apparently healthy vs. ill), since the etiology of hypoglycemia varies accordingly. In apparently ill adults, the most common causes include drugs and toxins (notably alcohol), organ failure (hepatic, renal, or cardiac), sepsis, and endocrinopathies (adrenal insufficiency and GH deficiency). In apparently healthy adults, causes of endogenous hyperinsulinism must be considered. The most frequent is insulinoma, although other less common conditions such as autoimmune disorders (anti-insulin or anti-insulin receptor antibodies), factitious hypoglycemia, post-bariatric surgery hypoglycemia, non-insulinoma pancreatogenous hypoglycemia syndrome, and rare familial syndromes should also be included. The gold standard to establish the underlying cause is the 72-h fasting test (Table 1) (4, 5).

Insulinoma is a rare tumor, but it is the most common cause of endogenous hyperinsulinism in the general population, with an annual incidence of 1–4 cases per million people. There is a slight female predominance (59%), and the mean age at diagnosis is 45 years in sporadic cases (in the context of multiple endocrine neoplasia, it typically occurs in the third decade of life). Most insulinomas are small (1–2 cm), unilateral, and benign, making the vast majority surgically curable (6–9).

Hypoglycemia after pancreas transplantation is very common, affecting up to 55% of patients (3, 9). CGM studies have improved our understanding of post-transplant carbohydrate metabolism. Hypoglycemia episodes usually follow a nocturnal pattern, occurring between 12 a.m. and 7 a.m., unrelated to postprandial periods. Most episodes are asymptomatic and resolve spontaneously without intervention (10, 11).

Determining the etiology of hypoglycemia in patients with SPK transplantation is particularly challenging due to the inherent hyperinsulinemic state associated with systemic venous drainage. Interpretation of the classical fasting test is further complicated by the absence of standardized reference values in this specific population. The pathophysiological mechanisms remain debated. Proposed explanations include reduced carbohydrate intake due to fear of hyperglycemia, peripheral hyperinsulinemia, defective counterregulatory responses, insulin autoantibodies prolonging insulin half-life, and abnormal pancreatic islet cell growth. Additionally, the duration of pre-transplant diabetes may be a risk factor, particularly in the setting of impaired glucagon secretion. Nevertheless, general causes of hypoglycemia should not be excluded and must be considered in the differential diagnosis (4, 12).

Managing hypoglycemia in pancreas transplant patients is therefore complex. First, it is crucial to differentiate expected post-transplant phenomena from pathological conditions requiring further evaluation. We propose a comprehensive, targeted clinical assessment complemented by CGM, which provides essential information regarding hypoglycemia patterns, duration, frequency, spontaneous recovery, and time spent in hypoglycemia. Second, the limitations of the fasting test must be acknowledged, and additional diagnostic tools should be incorporated. These include tumor markers (e.g., chromogranin A), conventional imaging (contrast-enhanced CT), and functional imaging (68Ga-DOTA-TOC PET-CT), highlighting the importance of a multidisciplinary diagnostic approach.

## Data Availability

The original contributions presented in the study are included in the article/Supplementary Material, further inquiries can be directed to the corresponding author.
